# Neurology, or the Doctrine of the Nerves*We have selected this portion of Dr. Buck well’s thesis, because it gives in a brief and condensed manner the distribution of the nerves most implicated in dental diseases—also, giving the distribution of the nerves of taste.

**Published:** 1851-07

**Authors:** E. Buckwell

**Affiliations:** Zanesville, Ohio


### DENTAL REGISTER OF THE WEST.

VOL. IV. JULY, 1851. NO. 4

### NEUROLOGY,

### OR THE DOCTRINE OF THE NERVES.*

EXTRACT FROM A THESIS PRESENTED TO THE FACULTY OF THE OHIO COLLEGE OF DENTAL SURGERY, FEBRUARY, 1851,

BY DR. E. BUCKWELL, OF ZANESVILLE, OHIO.

The nerves undoubtedly perform a very important part in the animal economy. Amidst the many important discoveries that persevering and scientific Physiologists have made in the laws that govern the animal body, still the knowledge of the functions of this mysterious system is enveloped in doubt and obscurity, while science and untiring industry has accomplished a great deal towards dispelling those crude theories with which this subject was formerly invested, and the researches of Bell, Magendie, Marshall, Hall, Carpenter and others, have contributed largely to our knowledge of the nervous system. Still much remains to be done.

The nerves constitute the immediate organs of sensation, and convey impressions made on them to the mind, although the manner in which these impressions are produced, and the means by which the will is conveyed from the brain to the muscles is still a matter of doubt, while there are some who suppose that sensation is conveyed to the brain by means of a

*We have selected this portion of Dr. Buckwell's thesis, because it gives in a brief and condensed manner the distribution of the nerves most implicated in dental diseases--also, giving the distribution of the nerves of taste.

vibration communicated to the nerve or by a nervous fluid contained in them. Others contend that it is by means of an electric agent common to them and many other substances. They are divided into motor and sentient nerves, while some are a compound of both these functions. They are invested by a sheath called neuralema. They are also distinguished as cerebral or cranial and spinal nerves. In some parts of the body they unite and form a plexus, a trunk, or a ganglion. The cerebral so called from their arising from the brain emerge through the foramina at the base of the cranium. They consist of nine pairs. (The eighth or pneumogastric has a double origin as it arises partly from the spinal cord opposite the * fourth cervical vertebra.) They are divided into three classes according to their functions. Nerves of special sense, including the first second and the auditory branch of the seventh. Nerves of motion including the third fourth sixth and facial branch of the seventh and ninth and part compound nerves comprising the eight and fifth.

Out of this collection the fifth or tri-gemini more particularly claims the attention of the Dentist, or rather that portion which issues from the ganglion of Casser which is upon the trunk of this nerve. From this ganglion proceeds three branches. The opthalmic the superior and inferior maxillary. The superior maxillary makes its exit through the foramen rotundum of the sphenoid bone, and then enters the pterygo-maxillary fossa. A large branch called infra orbital traverses the infra orbital canal and emerges at the infra orbital foramen to supply the face giving off the dental nerves in its course. The pterygo palatine branch descends through the canal leading to the posterior palatine foramen and running near the alveoli, sends branches to the velum palati and palate plate of the superior maxillary bone. It sends some filaments which pass round the upper jaw and vanish in the cheek;

* The eighth pair is composed of three parts, viz: the *par Vagum* or *Pneumogastric*, the Gloss-phayngeal, and spinal accessory of Willis. The root that originates the two first branches, arises from the medula oblongata, while that of the spinal accessory springs up from the spinal cord, opposite the fourth cervical vertebra.

others again which pass round the tuberosity of the superior maxillary bone, and supply its substance as well as the molar teeth and the membrane lining the antrum highmoranium. The spleno-palatine branch of the superior maxillary nerve, after sending a portion to join the great sympathetic in the carotid canal, and another which enters the foramen Innominatum to join the portia dura of the seventh pair of nerves, proceeds horizontally inwards through the spheno-palatine foramen, ontinuing along the septum contiguously to the floor, and through the anterior palatine foramen to the roof of the mouth, where it inosculates with the pterygo-palatine nerve. Small filaments are sent forward through the substance of the bone to the anterior incisor teeth. It is by these several branches of the superior maxillary nerve that the teeth of the upper jaw are supplied by nerves. The incisors receive theirs from branches sent forwards from the junction of the pterygo-palatine, and spheno-palatine, at the foramen incissivum. The canine and bicuspid receive theirs from branches sent down from the infra orbitar nerves and the molars are supplied by the pterygo-palatine and alveolar branches.

The inferior maxillary is the largest branch given off from the ganglion of Casser; it passes downwards and outwards through the foramen ovale of the sphenoid bone, then down on the inner side of the ascending ramus, between the pterygoideus externus and internus muscles, and passing between the lateral ligament and the bone, proceeds through the posterior mental foramen. It gives off four branches. A gustatory branch which passes between the two pterygoidei and proceeds to the tongue—a temporal branch which goes to the temporal muscle—a branch which emerges from the masseter, and passes along the buccinator. A small branch given off just as the nerve enters the posterior mental foramen, and passes along a groove in the bone to the mylo-hyoideus. The continuation of the nerve passes through the mental foramen, and gives branches upwards to the teeth. As it emerges from the anterior mental foramen, it communicates with the pes anserinus of the portia dura of the seventh pair. Physiologists describe the opthalmic and superior maxillary as sentient nerves, while the inferior maxillary is both sentient and motor.

The nerves of taste suggest considerations of marked importance to the practical Dentist, more especially in the mechanical branch. They consist of the lingual branch of the fifth. The eighth or glosso-pharyngial, and the ninth or hypoglossal. The lingual branch of the fifth, it is generally conceeded is first in the list of the nerves of taste. It supplies the front and dorsal surface of the tongue, as well as the Papillae at the apex. The glosso-pharyngial is also a sentient nerve. According to Dr. Carpenter's description, it is principally distributed on the mucous surface of the fauces and on the back of the tongue, but sends a branch forward to the inferior surface of the tip, which branch anastomoses with the lingual nerve. Dr. Carpenter is disposed to regard the latter as the medium of the sense of taste as well as touch in those parts of the tongue to which it is especially distributed. This mutual function serves to explain why the tongue—the arches of the palate—the tonsils, and uvula, all possess the sense of taste, though in a varied degree. The ninth or hypoglossal is chiefly the motor nerve of the tongue.

Nerves are liable in common with other organs of the body to functional derangement. Diseases of the nerves are termed neuralgic. The orbitar branch of the fifth pair is the one most frequently affected with neuralgia, and the division of this branch immediately below the Infra orbitar foramen used to be resorted to for the cure of that painful affection. Of late that operation is less frequently performed. The malady being generally attributed to constitutional causes, consequently constitutional treatment is more generally had recourse to. One especial reason for this is the fact that the nerves like other parts of the frame, are capable not only of being repaired but even reproduced after an injury, and after a nerve has been completely divided and its functions totally suspended, it may gradually resume its power, and like a severed muscle or tendon the ends may become again connected by the formation of a new substance, although it may reasonably be questioned whether the new formation is ever so completely organized as the original. We are warranted in presuming it is not, from the fact that it is not the case with other organs of the body. Persons of a nervous temperment are more subject to this painful affection than those of a lymphatic. Hence any cause that will affect the mind, mental anxiety, close study, loss of property or near and dear friends, may operate to produce this malady. Although this affection is more commonly of a constitutional character, it frequently assumes a local form. A twig of the nerve being sometimes the seat of the malady, the main body of the nerve being acted on merely by sympathy or through the medium of local inflamation. Pressure arising from inflamation of surrounding parts—from some foreign substance or from a morbid growth, as for example, an exostosis of the apex of the root of a tooth, another cause arising from inflamation as connected with the teeth, may be seen in the distension of the blood vessels in the dental canal pressing on the nerve accompanying them. The treatment of diseases of the nerves would of course depend on whether the affection was constitutional or local, and would be more within the province of the physician than the dentist. At this stage of the matter I am admonished by the recollection of the opinion given by one of old, "That a fool multiplies words."—I have done.

